# Local oestrogen therapy modulates extracellular matrix and immune response in the vaginal tissue of post‐menopausal women with severe pelvic organ prolapse

**DOI:** 10.1111/jcmm.14199

**Published:** 2019-02-17

**Authors:** Tanya Tyagi, May Alarab, Yvonne Leong, Stephen Lye, Oksana Shynlova

**Affiliations:** ^1^ Department of Physiology University of Toronto Toronto ON Canada; ^2^ Lunenfeld‐Tanenbaum Research Institute Mount Sinai Hospital Toronto ON Canada; ^3^ Department of Obstetrics & Gynecology University of Toronto Toronto ON Canada; ^4^ Division of Urogynecology and Reconstructive Pelvic Surgery Mount Sinai Hospital Toronto ON Canada

**Keywords:** oestrogen, local oestrogen therapy, POP

## Abstract

This study investigates the effect of local oestrogen therapy (LET) on the expression of proteins participating in collagen/elastin biogenesis and immune markers in vaginal tissues of post‐menopausal women with severe pelvic organ prolapse (POP). Vaginal biopsies were collected from the anterior vaginal wall of informed and consented 52 post‐menopausal women with severe POP undergoing total hysterectomy. Twenty‐nine of the 52 women were treated with LET (in the form of vaginal oestrogen cream or tablet), while the remaining 23 untreated patients served as the controls. This study was approved by Sinai Health System REB. Vaginal tissue specimens were analysed for gene and protein expression using real‐time RT‐PCR and Luminex assays, protein localization and immune cell infiltration were assessed by immunohistochemistry. Forty‐four cytokines were detected. We found that LET application: (a) significantly increased (*P* < 0.05) gene and protein expression levels of extracellular matrix (ECM) structural proteins, collagen and elastin, as well as the expression of ECM maturation enzyme *BMP1*; (b) decreased protein expression level of ECM degradation enzymes MMP1, MMP2 and MMP3 accompanied by an increase in their tissue inhibitors, TIMP1 and TIMP4; (c) significantly increased (*P* < 0.05) the gene and protein expression levels of 14 vaginal cytokines involved in leucocyte infiltration, which was confirmed by immunohistochemistry. Our results indicate that LET plays an important role in the activation of immune system within the local vaginal environment, limiting the undesirable ECM degradation, which supports the strengthening of vaginal ECM in post‐menopausal women, therefore resisting menopause/age‐related changes and inducing urogenital tract tissue regeneration.

## 1 INTRODUCTION

Pelvic organ prolapse (POP) represents a major health issue for women worldwide and affects nearly 50% of the population of post‐menopausal women.^1^ Multiple risk factors that lead to POP also include race, parity, obesity and chronic conditions (cough, constipation) that result in higher pressure on the pelvic floor. POP is the most common reason for hysterectomy among post‐menopausal women.^2^, ^3^ Management options for POP include observation, pessary use and urogynaecologic surgery. The lifetime risk for undergoing at least one operation for POP is 11.1%, however, the re‐operation rates are as high as 29%. With an ageing population, the demand for POP operations is expected to rise in the future.^2^

Local oestrogen therapy (LET) is clinically used as an aid in POP treatment. In particular, as hypoestrogenism is a major risk factor for POP, some expert gynaecologic surgeons recommend pre‐ and/or post‐operative vaginal oestrogen therapy for post‐menopausal women.^3^, ^4^ Importantly, the severity of POP symptoms has been shown to increase after menopause, which suggests that hypoestrogenism may be a contributory factor for the development of POP.^5^, ^6^ However, there is no consensus in the medical community about the efficacy of prescribing LET to patients with prolapse leading to a difference in patient care. There are few studies available, which directly address the effect of vaginal oestrogen on POP to ascertain the theoretical basis for its therapeutic application. Circulatory levels of oestrogen in females change in a monthly cyclic pattern during reproductive years. At menopause, however, oestrogen levels in the blood decrease significantly from 15‐350 pg/mL (menstruating women) to 6.5 pg/mL (post‐menopausal women).^7^, ^8^ Deficiency in oestrogen following menopause affects reproductive organs, causing vaginal dryness, and reduction in vaginal weight.^9^ These vaginal and urinary symptoms are defined as the genitourinary syndrome of menopause (GSM). Oestrogen plays a supportive role in the pelvis by controlling the synthesis and breakdown of collagen.^10^ Oestrogen receptors α and β (ESR1/2) are present throughout the lower urinary tract, bladder and vagina; with ESR1 being the predominant isoform.^11^ They are expressed in three vaginal layers (epithelium, lamina propria and muscularis), and are responsive to steroid hormones.^12^ Oestrogen therapy has been prescribed to treat urinary symptoms related to urogenital atrophy, such as vaginal dryness, irritation or itching, dyspareunia and thin and frail epithelium.^15^ ESRs are also found on a variety of immune cells through which estradiol plays an important role in the regulation of the immune system.^13^, ^14^

Additionally, studies have reported a connection between the development of POP and abnormalities in connective tissue structure and repair mechanisms, such as in generalized connective tissue disorders like Ehlers Danlos or Marfan syndrome.^16^ We have previously shown that the weakened pelvic floor may arise from aberrant biosynthesis and biodegradation of structural proteins of the connective tissue. POP patients have lower levels of structural proteins, and maturation enzymes, including lysyl oxidase (LOX) family of enzymes, ADAMTS2 (A Disintegrin and Metalloproteinase with Thrombospondin motifs 2, also known as PNP) and bone morphogenetic protein‐1 (BMP‐1, also known as PCP), and higher degradation activity with an increase in matrix metalloproteases (MMPs) expression and activity and a decrease in tissue inhibitors of MMP (TIMPs).^16^, ^17^ Importantly, there is extensive literature affirming that the immune system plays a role in extracellular matrix (ECM) regulation. For instance, mice deficient in T and B cells exhibit decreased total and cross‐linked collagen and LOX, and at the same time, increased MMP2, suggesting a role of lymphocytes in ECM remodelling.^18^

In pre‐menopausal women, hormonal regulation affects the expression of multiple genes regulating immune cell composition in the vagina, and endocrine balance in the mucosal immune system.^19^, ^20^ However, this type of regulation is not present in post‐menopausal women. Changes in the immune system accompanying ageing and oestrogen deprivation are known as immunosenescence, the process characterized by a decrease in cell‐mediated immune function and humoral response^21^ which is reflected by a higher incidence of chronic infectious disease in post‐menopausal women.^22^ Studies have shown that hormone replacement therapy (HRT) in post‐menopausal women can improve wound healing, modulate cytokine production and proliferation of immune cells.^23^, ^24^ It appears that oestrogen plays an important role in restoring altered immune profile with normal ageing peri‐menopausal women, suggesting that preservation of immune function may be supported by systemic HRT. Despite these known effects of oestrogen, not much work has been done to examine the effect of LET on the immune system of patients with POP. Thus, the objective of this study was to investigate the effect of LET (oestrogen cream or tablet) on the expression of proteins participating in collagen/elastin biogenesis and on modulation of immune response in vaginal tissues of post‐menopausal women with severe POP.

## 2 MATERIALS AND METHODS

### 2.1 Patient selection

The Research Ethics Board of Mount Sinai Hospital, Toronto approved this study (05‐0193‐E). Vaginal biopsy samples were collected from post‐menopausal women undergoing vaginal hysterectomy for POP following informed consent. All patients had grade 3‐4 of prolapse as per POP‐Q classification. The POP patients were either receiving local oestrogen therapy (LET group, n = 29) through oestrogen cream (Premarin) or tablets (Vagifem) or were not receiving oestrogen therapy (No‐LET control group, n = 23). Premarin cream was taken once daily (0.5 g) for 10 days, then twice weekly continuously until surgery; Vagifem tablet was taken one tablet once daily for 2 weeks (10 μg) then twice weekly until surgery day. Women with a history of gynaecological malignancy, connective tissue disorders were excluded.

### 2.2 Tissue collection

The tissue biopsy technique used in this study has been described previously.^25^ Full‐thickness vaginal wall biopsy (approximately 1 cm^2^) was obtained from the anterior middle portion of the vaginal vault and placed in ice‐cold PBS solution. One part of the vaginal biopsy was fixed in formalin for histological and immunohistochemical analyses, the second part was flash‐frozen in liquid nitrogen and stored for biochemical assays (RNA extraction for gene expression analysis, proteins extraction for Luminex and enzyme‐linked immunosorbent assay [ELISA]). Due to some variability in the size of the sample, sometimes tissue biopsy was used only for one application.

### 2.3 RNA extraction & reverse transcription

Frozen tissue samples were crushed and total RNA was extracted using Trizol (Gibco, Canada) according to the manufacturer's protocol. RNA samples were purified using RNeasy MiniElute Cleanup Kit (Qiagen, Canada) and treated with 2.5 μL DNase I (Qiagen) to remove genomic DNA contamination. Total RNA was then reverse transcribed to cDNA (1 μg of RNA in 20 μL reaction) using iScript supermix (Bio‐Rad, Canada) to produce cDNA at a final concentration of 50 ng/μL.

### 2.4 Real‐time RT‐PCR

Five nanograms of cDNA was mixed with SYBR^®^ green PCR ready mix (Sigma‐Aldrich, Canada) and specific primer pairs (Table 1). Reverse transcription (RT)‐PCR was performed with the CFX384 Touch^™^ RT‐PCR detection system. A no‐template control was used to control for any contaminations in a mastermix. PCR reactions were set up in triplicates and the cycle threshold (Ct) value was recorded for each primer pair by CFX Manager software (Bio‐Rad). The expression level of target genes was normalized to the geometric mean of three housekeeping genes: TBP, SDHA and YWHAZ, and a comparative Ct method (∆∆CT) was used to get relative gene expression values. All mRNA levels for LET‐treated post‐menopausal POP patients were expressed as fold changes relative to mRNA levels in the vaginal tissue of the control non‐LET‐treated POP patients.

**Table 1:** Real‐time PCR primer sequences of a panel of human genes encoding collagens, elastin and elastin‐related proteins, enzymes involved in their maturation (LOX family) and cytokines

Symbol	Accession no.	Forward	Reverse
COL1	NM_000088.3	GCACCATCATTTCCACGAGC	GTCAGATGGGCCCCCG
COL3	NM_205380.2	GGTAGTCTCACAGCCTTGCG	GAGGATGGTTGCACGAAACAC
COL4	NM_001845.5	TGGGAAACCTTTTGGGCCTG	TAGGCACAGGACCTTTGGGA
COL5	NM_000093.4	TCCGAAGGGGCCAGAATCA	GAGCAGTTTCCCACGCTTGA
ADAMTS2	NM_014244.4	GTGTGCACCTGGCAAGCATTGTTT	AGCCAAACGGACTCCAAGCGC
BMP1	NM_001199.3	GCCACATTCAATCGCCCAA	TGGCGCTCAATCTCAAAGGAC
LOX	NM_002317.6	GGCGAAGGGTGAGGAGTAAG	TGGGAGACCTAAACGTCAGC
LOXL1	NM_005576.3	TATGTCCAGAGAGCCCACCT	TAGCACCCGCACATCGTAGT
LOXL2	NM_002318.2	ATGTCACCTGCGAGAATGGG	TGCTCTGGCTTGTACGCTTT
LOXL3	NM_001289164	TCAGCCAGAAAGGCAAGGAG	GGGGACGAGAAACACTGACC
ELN	NM_000501.3	CCTCCCTTCTGCTTCCTCTC	CGACTGTTCTTTCGCTGCTG
FBN1	NM_000138.4	TCTGCACAAAAACGCTCTGC	CTCCCGTGCGGATATTTGGA
FBN2	NM_001999.3	CTCTTCTTCTGGGGGCGACTT	CGCTCCGAAGACGGATATTGG
FBLN5	NM_006329.3	TCTGGAAAGGGCAGCAACTT	CTTGTCTATCAGCCGATGCG
MIF	NM_002415.1	CTCCACCTTCGCCTAAGAGC	TTCTCCCCACCAGAAGGTTG
IL16	NM_004513.5	TCTACAGCAGAGGCCACAGT	GAGGCTTGTCTCCGTGTAGG
IFN‐y	NM_000619.2	TCAAACCGGCAGTAACTGGAT	GGCAGCCAACCTAAGCAAGA
CX3CL1	NM_002996.4	CGGTGTGACGAAATGCAACA	CCGCATGATGCCTGGTTCT
CCL3	NM_002983.2	TCCGTCACCTGCTCAGAATCA	GATGCAGAGAACTGGTTGCAGA
CXCL12	NM_199168.3	CTTTCCGCTAGACCCACTCG	CTCATGGTTAAGGCCCCCTC
IL1b	NM_000576.2	CCATCAGCCAGGACAGTCAG	TCAGGCGGGCTTTAAGTGAG
CCL21	NP_002980.1	CTGGACAAGACACCATCCCC	CTCAGTCCTCTTGCAGCCTTT
IL8	NM_000584.3	AAACATGACTTCCAAGCTGGCCGT	GCAAAACTGCACCTTCACACAGAGC
IL6	NM_000600.4	TGGCTTGTTCCTCACTACTCT	TCAATGAGGAGACTTGCCTG
CXCL9	NM_002416.2	TAAGCGCTAGAGGAAGCAGC	CCCTGGAAGGAGGTTTCCAC
MIP1b	NM_002984.3	GTCTGTGCTGATCCCAGTGA	CAGGTGACCTTCCCTGAAGAC
TBP	NM_003194.4	CCACAGCTCTTCCACTCACA	CTGCGGTACAATCCCAGAAC
YWHAZ	NM_003406.3	CCGCCAGGACAAACCAGTAT	ACTTTTGGTACATTGTGGCTTCAA
SDHA	NM_004168.3	CCACCACTGCATCAAATTCATG	TGGGAACAAGAGGGCATCTG

### 2.5 Luminex protein assay

Frozen vaginal tissue samples (n = 19 per group) were crushed and total protein was extracted using Bicine lysis buffer (Sigma‐Aldrich). Tissue MMPs, TIMPs and cytokine levels were quantified in vaginal tissue samples (500 μg) using Bio‐Plex Pro^™^ Human 9‐Plex MMP assay, Human 4‐plex TIMP assay and Bio‐Plex ProTM Human Cytokine Assays (40‐plex) in combination with a custom designed 5‐plex (all Bio‐Rad), following manufacturer's instructions. The standard and sensitivity of all cytokines used are reported in Table S1. Tissue samples, standards and controls were analysed in duplicates. The plates were read on Bio‐Plex^®^ 200 System instrument with high‐throughput fluidics and analysed using the Bio‐Plex ManagerTM 5.0.

### 2.6 Enzyme‐linked immunosorbent assay

Human IL‐1RA Duoset Elisa kit (R&D Systems, Minneapolis, MN, USA) was used to measure protein expression levels in human vaginal tissues (N = 19/group), as instructed by the manufacturer's protocol. Based on the standard range of the ELISA kit, tissue samples were diluted to ensure the absorbance of readings stayed within the linear range of standard curve. The plate was read using μQuantTM (BioTek^®^ Instruments Inc.) with wavelength settings specified by the ELISA kit manufacturer.

### 2.7 Histochemical analysis of total collagen and elastin

Vaginal tissue biopsy samples fixed in formalin were embedded in paraffin, cut into 5 μm slides and stained with a modified Masson's Trichrome stain to measure the total protein content of collagen and elastin. Verhoff's haematoxylin stain was used to stain elastic fibres black and Biebrich scarlet‐acid fuchsin was used to stain collagen green‐blue. All slides were scanned at 400× magnification, and pictures were imported into Visiopharm NewCast Software (version 6.6.1.2572) EngineTM for quantification. The operator was blinded to the treatment groups to prevent bias. The ‘Region of Interest’ was identified on the individual vaginal tissue sample (Lamina Propria, LP or Muscularis layer, M) using a masking tool and the total area was recorded in two replicates for same biological sample. The section was divided into smaller sampling areas (n = 20 per region of interest) using a grid consisting of uniformly spaced points. The area of total collagen and total elastin was measured within each sampling area by number of grid points overlying on collagen [blue staining] or elastin [black staining] and compiled to give the total area for collagen and elastin for each tissue. The relative amount of collagen and elastin for each tissue was calculated as the ratio of total area for collagen or elastin to the total area of region of interest (relative collagen content = total collagen area in tissue/area of tissue; relative elastin content = total elastin area/area of tissue). These relative quantities were compared between the LET and Control (No‐LET) groups (n = 10/group).

### 2.8 Immunohistochemistry

Tissue samples were fixed in 10% neutral buffered formalin or 4% paraformaldehyde and embedded in paraffin. Following rehydration and quenching (3% H_2_O_2_), antigen retrieval was performed by microwaving slides in 10 μM sodium citrate solution for 20 minutes (pH 6). Following the retrieval, slides were blocked using protein blocking solution (DAKO) and then incubated overnight at 4°C with primary antibody (Table 2). For a negative control, non‐specific rabbit IgG (DAKO) was used at the same concentration as primary antibody. Next, sections were incubated with biotinylated secondary antibodies followed by Streptavidin‐HRP. Finally, tissue sections were mounted with Surgipath Micromount^®^ mounting media (Leica Microsystems Inc.) and examined on an Olympus BX61 Motorized Microscope and MicroSuite^™^ system (Olympus America Inc.). The operator was blinded to the treatment groups to minimize operator bias. A minimum of five fields were examined and photographed for each tissue section using Olympus DP72 Microscope Digital Camera (Olympus America Inc.). The number of positively stained immune cells were counted by the blinded operator and recorded to quantify the infiltration of immune cells into the tissue.

**Table 2:** Summary of antibodies used in immunohistochemical analyses

	Antibody	Dilution	Source	Company
SMA	Monoclonal	1:50	Rabbit	Abcam
Collagen I	Polyclonal	1:100	Rabbit	Abcam
Collagen III	Polyclonal	1:1000	Rabbit	Abcam
Collagen V	Polyclonal	1:50	Rabbit	Abcam
CD45	Monoclonal	1:200	Mouse	Dako
CD68	Monoclonal	1:200	Mouse	Dako
Anti‐rabbit IgG	Polyclonal	1:200	Goat	Dako
Anti‐mouse IgG	Polyclonal	1:200	Goat	Dako

### 2.9 Statistical analysis

Statistical analysis was performed with independent sample *t* test for continuous data and chi‐squared test for categorical data. Graphs were presented as mean ± SEM. The level of significance was set at *P* < 0.05 (*), *P* < 0.01 (**), and *P* < 0.001 (***). GraphPad Prism version 5.0 was used for all statistical analysis (GraphPad Software Inc, USA). For experiments involving a subset of all samples collected, an online random number generator was used to select for the samples without bias.

## 3 RESULTS

### 3.1 Patient demographics

Fifty‐two post‐menopausal women with severe POP were recruited into the study. Twenty‐nine of these women were using LET, while 23 women did not undergo any hormonal therapy (Control, No‐LET). Groups were matched for age (mean age 64 vs 67 years), mean BMI (26.2 vs 26.1) and median parity (2 vs 2). The majority (79%) of the women recruited for this study had Stage III prolapse, with some (21%) with Stage IV prolapse. The duration of LET for the post‐menopausal women ranged from 1 to 24 months, with an average of 8 months (Table 3).

**Table 3:** Summary of patients’ demographics

Study groups	No‐LET	LET
n	19	19
Mean age	65 ± 7	66 ± 7
Mean BMI	25.8 ± 3.7	25.5 ± 3.8
Median parity	2 (1‐5)	2 (1‐7)
Duration of LET (month)	0	8 (1‐24)^a^
Stage of POP (n)	Stage III (n = 16) Stage IV (n = 3)	Stage III (n = 14) Stage IV (n = 5)

*T* test; level of significance: *P* < 0.05.

BMI, Body mass index; POP, pelvic organ prolapse.

a Statistical significance between No‐LET and LET groups.

### 3.2 Effect of LET on ECM biosynthesis and biodegradation

**Figure 1:**
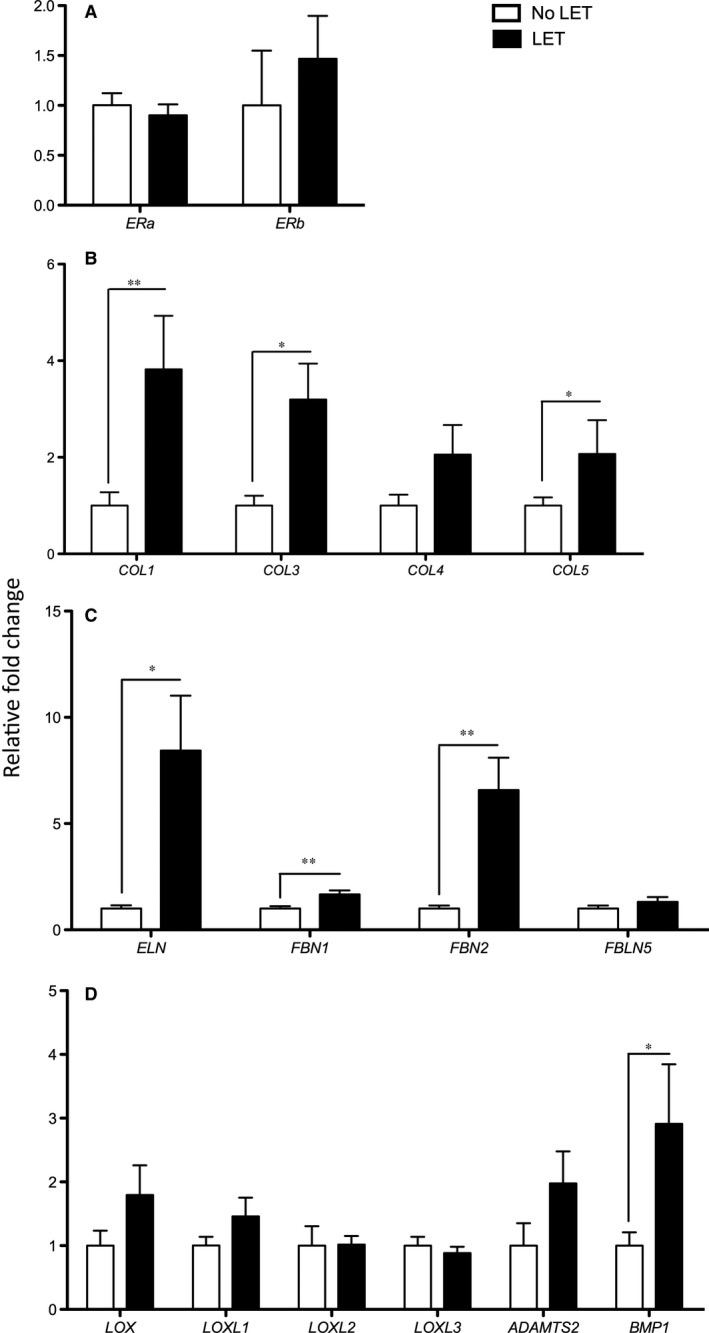
Gene expression of extracellular matrix genes in vaginal biopsies of post‐menopausal POP patients. A, Transcript levels of Oestrogen Receptors (*EST1, EST2*), (B) Collagens (*COL1, 3, 4, 5*); (C) Elastin (*ELN*) and Elastin‐related proteins (*FBN1, FBN2, FBLN5*); (D) ECM maturation enzymes (*LOX, LOXL1‐4, ADAMTS2, BMP1*). Data presented as fold change in LET‐treated group (black bars, N = 29) compared to no LET (control, white bars, N = 23). Statistical analysis was performed with independent sample *t* test. A significant difference is indicated by * (*P* < 0.05), ** (*P* < 0.01)

Transcript levels of ECM structural and remodelling genes: collagens (*COL1,3,4,5*), elastin (*ELN*), proteins involved in elastin assembly fibrillin1 and 2 (*FBN1, FBN2)* and fibulin5 (*FBLN5)*, ECM maturation enzymes (*LOX, LOXL1‐3, BMP1* and *ADAMTS2),* and oestrogen receptors (*ESR1, ESR2*) were examined. We found the presence of mRNA of both oestrogen receptor isoforms (*ESR1* and *ESR2*) in human vagina. There was no significant difference in the expression of either transcript between LET vs No‐LET groups, indicating that LET does not influence ESR receptor level (Figure 1A).

Among the genes encoding ECM structural proteins, the expression level of three major structural collagens (*COL1, COL3, COL5*) showed an increase in the mRNA levels in the vaginal tissues of LET patients as compared to No‐LET POP patients (Figure 1B, *P* < 0.05). *COL4* transcript levels were increased in LET‐treated tissues, however the difference did not reach significance (*P* = 0.076). In addition, there was a significant increase in the expression of *ELN* gene after treatment with LET (Figure 1C, *P* < 0.05). Transcript levels of proteins involved in the deposition and maturation of elastin fibres, *FBN1* and *FBN2* showed a significant increase, while *FBLN5* showed no significant difference between the two study groups (Figure 1C, *P* = 0.24). The gene expression level of ECM maturation enzyme *BMP1* showed a significant increase (*P* < 0.05) in LET as compared to No‐LET group. In contrast, expression of *ADAMTS2* and LOX family members (*LOX, LOXL‐1,2,3*) did not differ significantly between the two groups (Figure 1D).

### 3.3 Immunohistochemical localization of ECM proteins

**Figure 2:**
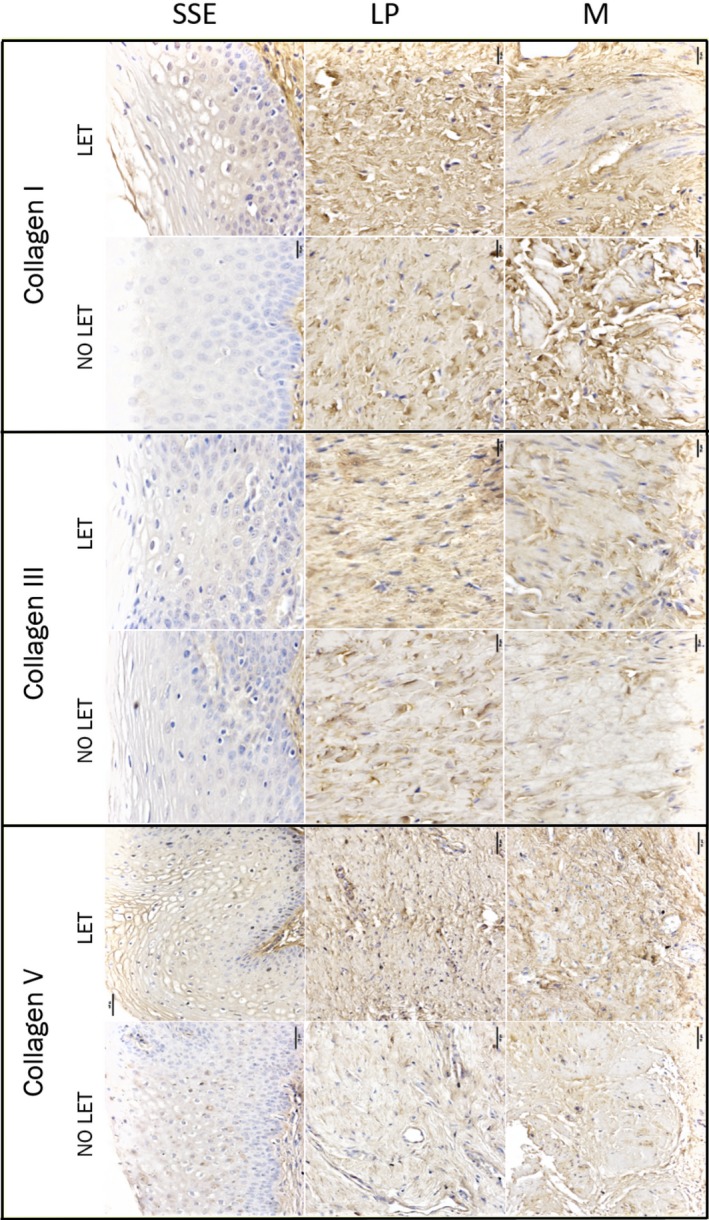
Immunohistochemical localization of Collagen I, III, and V within human vaginal biopsy samples of post‐menopausal women with severe POP. Shown are representative images of vaginal tissues from POP patients treated with LET and no‐treated controls. The immunolabelling for collagen proteins is indicated by brown deposit. Magnification is 200×; scale bar 50 μm. Negative control is shown on Figure S1

Firstly, using histologic haematoxylin, eosin staining and *α*‐smooth muscle actin (ACTC1) immunostaining, we confirmed the morphology of vaginal biopsy which included three anatomical layers: stratified squamous epithelial (SSE), LP and muscularis (M). The representative images for each stain are shown at Figure S1. Next, specific antibodies for ECM structural proteins (anti‐Collagen I, III and V) were used to establish their tissue localization on full‐thickness vaginal biopsies (n = 5 per group). The immunolabelling shows that the majority of collagens were localized within the lamina propria, some staining was detected in the muscularis layer in between the muscle bundles, however, no collagen immunostaining was detected in the SSE layer (Figure 2).

### 3.4 Total Collagen and Elastin content evaluation

**Figure 3:**
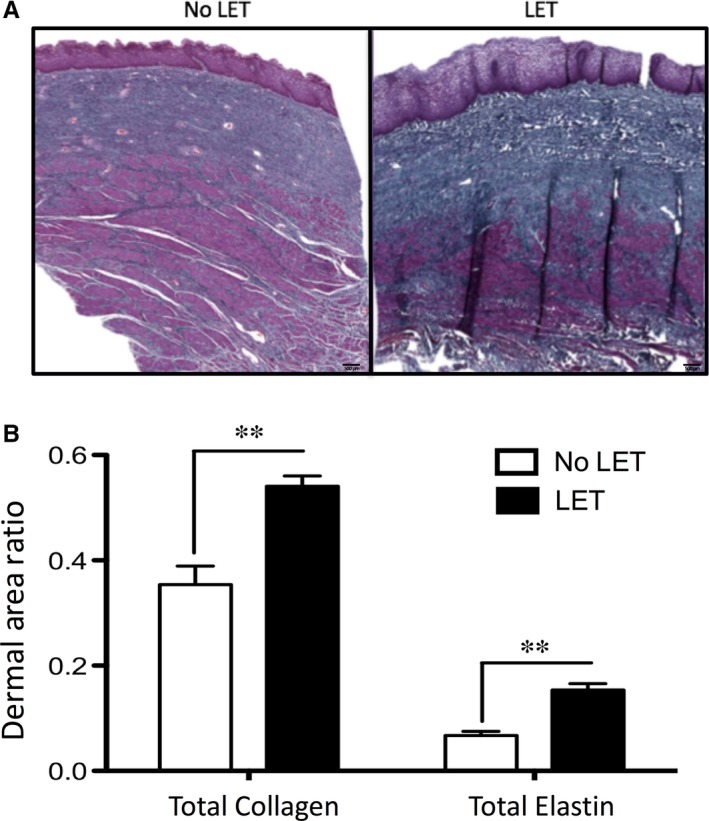
Modified Masson Trichrome staining for visualization of total Collagen and Elastin in vaginal tissues of patients with severe POP. A, Representative sections of vaginal biopsies showed expression and localization of total collagen (in blue) and elastin (in black) proteins. Magnification is 20×; scale bar 500 μm. B, Quantification of total Collagen and Elastin in vaginal biopsy samples from patients treated (black bars) and non‐treated with LET (white bars), N = 5/group. Data presented as ratio of area of collagen or elastin to the area of ROI (*P* < 0.05). A significant difference is indicated by ** (*P* < 0.01)

The modified Masson's Trichrome staining showed the expression and localization of total collagen (blue) and elastin (black) proteins in vaginal tissues, in particular their distribution across two layers of vaginal biopsies (i.e. LP and M), but not in the SSE layer (Figure 3). Quantification of the total protein content for both structural molecules showed that LET caused a significant increase (*P* < 0.05) in the content of collagen and elastin (1.53‐ and 2.29‐fold change respectively) in vaginal samples of post‐menopausal women with severe POP.

### 3.5 Effect of LET on ECM degradation enzymes

**Figure 4:**
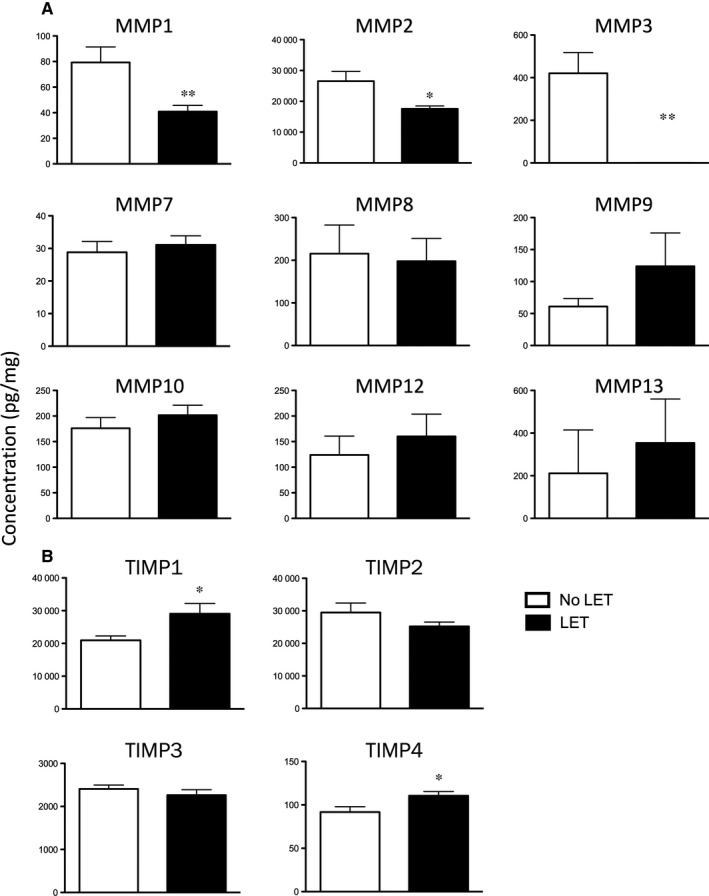
Protein concentration of MMPs (A, 9‐plex Luminex panel) and TIMPs (B, 4‐plex Luminex panel) in vaginal tissues of post‐menopausal women with severe POP treated with LET (black bars) and not treated (controls, white bars). N = 19/group. Data presented as mean ± SEM. Statistical analysis was performed with independent sample *t* test. A significant difference is indicated by * (*P* < 0.05), or ** (*P* < 0.01)

We analysed protein tissue lysates extracted from vaginal biopsy samples of post‐menopausal POP patients by high‐throughput approach, bead‐based 9‐plex MMP assay and 4‐plex TIMP 1‐4 assay (Bio‐Rad) on the Luminex 200 platform. An equal amount of total proteins (500 μg), was used to test the expression levels of multiple MMP and TIMP proteins. Data sets with extrapolated concentration values outside the limit of the standard curves were excluded from further analysis. All nine MMP proteins were detected. As we reported previously,^25^ of the four MMP inhibitors, TIMP1 and TIMP2 protein levels showed the highest abundance in vaginal tissue from patients with severe POP. TIMP4 expression was very low in human vagina. Samples from LET‐treated patients showed a significant decrease in the levels of MMP1, 2 and 3 proteins (Figure 4A, *P* < 0.05) and a significant increase for TIMP1 and TIMP4 (*P* < 0.05), while MMP7‐10, 12 13, as well as TIMP2 and 3 showed no change in protein expression in response to oestrogen (Figure 4B).

### 3.6 Effect of LET on cytokine protein expression

**Figure 5:**
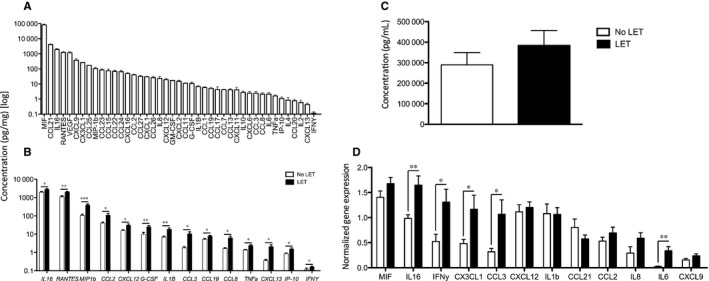
A, Expression profile of 44 cytokines detected by Luminex assay in human vaginal tissue from post‐menopausal women with severe POP. B, Comparative expression of 14 cytokine proteins in the LET‐treated group (black bars) and non‐treated group (control, white bars). Concentration of chemokines was determined in 500 μg of vaginal tissue lysates. A significant difference is indicated by * (*P* < 0.05), ** (*P* < 0.01), or *** (*P* < 0.001). C, ELISA analysis of IL‐1RA expression (pg/mL). No significant difference was detected between the two groups (*P* = 0.34). D, Analysis of cytokine gene expression between LET‐treated (black bars) and non‐treated (control, white bars) vaginal tissue samples. The expression of cytokine genes was normalized to three housekeeping genes: TBP, YWHAZ, SDHA. Statistical analysis was performed with independent sample *t* test. Data presented as mean ± SEM, N = 12

Next, the expression of 45 cytokine proteins was analysed in vaginal tissue samples from post‐menopausal POP patients and 44 cytokines were detected (Figure 5A and Table S2). All of the detected cytokines/chemokines play important roles in innate and adaptive immunity by inducing the migration or differentiation of immune cells. Interestingly, macrophage inhibitory factor/MIF had the highest level of expression among all chemokines studied in the panel (excluding IL‐1RA) with concentrations as high as 0.4 mg/mg of total protein (439 023.1 ± 53 111.5 pg/mg). CCL21 (chemoattractant for activated T cells) showed the highest levels of expression among CC family of chemokines (21 570.8 ± 2852.0 pg/mg). Interleukin (IL)‐16 (activator for T cells) also showed high levels of expression (1972.0 ± 193.4 pg/mg), while IL‐2, IL‐10, IL‐4, CCL20, CXCL13 and IFN‐γ proteins were detected at very low levels.

Next, we compared the expression levels of the above cytokines between LET‐treated and No‐LET women. Among the 44 detected cytokines, 14 showed a significant increase in LET‐treated samples: CCL2/MCP1, CCL3/MIP1a, CCL4/MIP‐1B, CCL5/RANTES, CCL8, CCL19, IL‐8/CXCL8, IP‐10/CXCL10, CXCL12, CXCL13, IL‐1B, IL‐6, IL‐16, G‐CSF, TNF‐α and IFN‐γ (*P* < 0.05, unpaired *t* test) (Figure 5B). These chemokines play important roles in the innate and adaptive immunity within vaginal tissue by mediating the inflammatory response (IL‐16, IFN‐γ, IL1B, TNF‐α), or inducing chemotaxis of monocytes/macrophages (MIP1b, IFN‐γ, IL‐16, IP‐10, CCL2, CCL3, CCL5, CCL8, CXCL12), T cells (IL‐16, CCL2, CCL8, CXCL10, CXCL12), B cells (CXCL13, CXCL12, CCL19) and granulocytes (IL‐16, CXCL8, CCL8). Some play a significant role in the induction of other chemokines, such as IFN‐γ, which can activate macrophages that produce IL1β, or induce CXCL9, one of the most highly expressed chemokine in vaginal tissue.

The expression levels for IL‐1RA were above the range of the standard in the Luminex assay and therefore could not be compared. IL‐1RA is an anti‐inflammatory protein, an important member of interleukin 1 cytokine family and plays a major role in dampening inflammation within different tissues in human body by antagonizing the role of IL‐1 and IFN‐γ. ELISA quantified the expression levels of IL‐1RA between two study groups which were not significantly different (383 340.4 ± 73 584.2 pg/mL vs 289 232.3 ± 60 139.42 pg/mL, *P* = 0.34) (Figure 5C). To confirm that LET also influenced transcriptional expression of vaginal cytokines, we extracted total RNA from vaginal biopsy tissues and analysed the transcript levels of cytokines that showed high levels of protein expression and a significant change in between the LET and No‐LET groups (Figure 5D). All tested genes were detected by RT‐PCR. Matching with Luminex results, IL16, IFN‐γ, CX3CL1 and CCL3 showed a significant increase in gene expression levels in LET‐treated samples (Figure S2).

### 3.7 Immunohistochemical assessment of leucocyte infiltration

**Figure 6:**
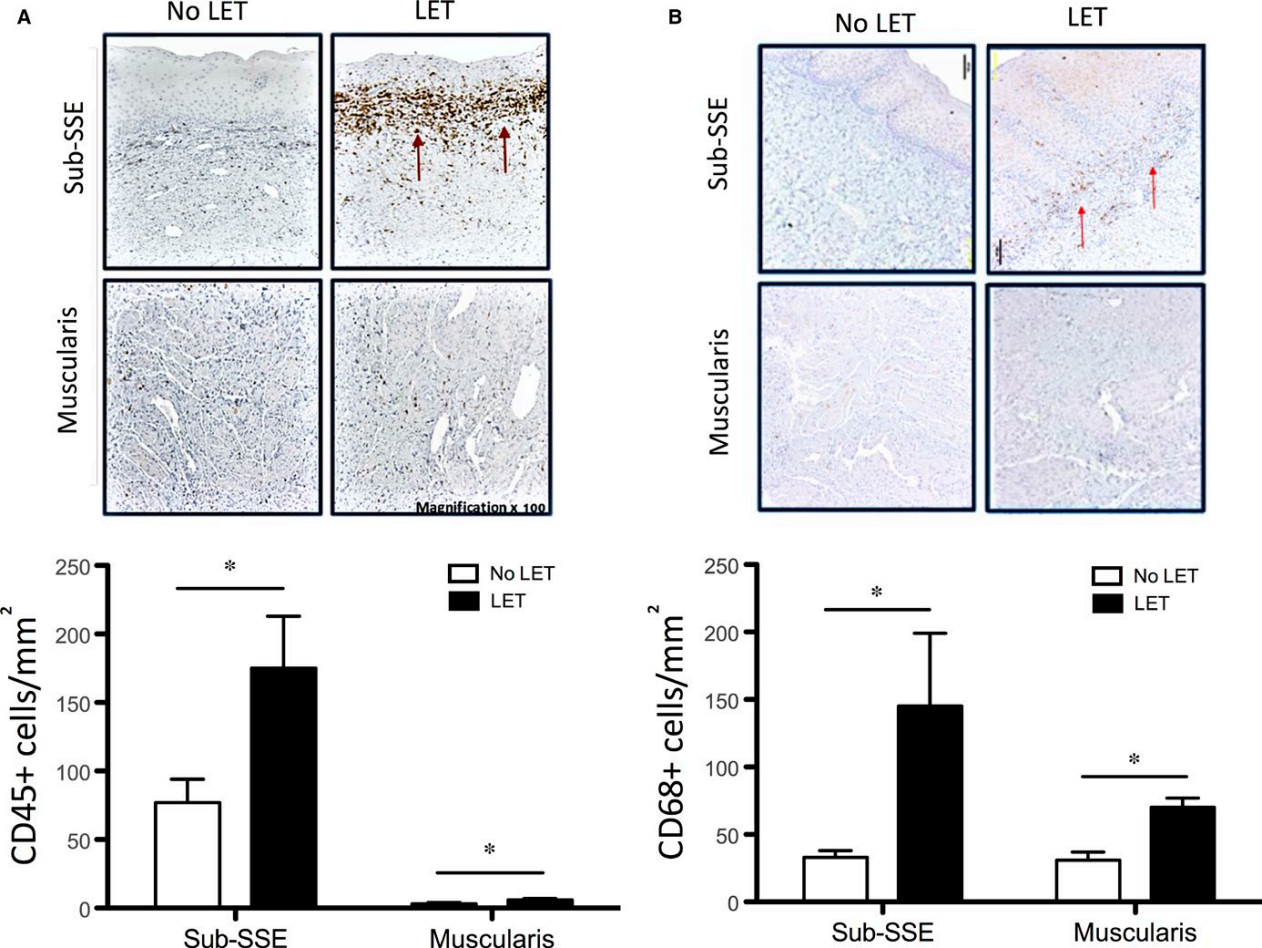
Infiltration of (A) leucocytes and (B) macrophages into vaginal tissues of post‐menopausal women with severe POP. There was a significantly (*P* < 0.05) higher number of CD45+ leucocytes and CD68+ macrophages (stain brown) in sub‐epithelial space (Sub‐SSE) and muscularis layer of human vagina in women treated with LET (black bars) as compared to control women (white bars). Magnification 100×; scale bar 100 μm. N = 10/group. A significant difference is indicated by * (*P* < 0.05)

As the vast majority of chemokines up‐regulated by LET are chemoattractants for immune cells, we next investigated whether they can induce the infiltration of leucocytes into human vaginal tissue. As we suggested, immunohistological staining for pan‐leucocyte marker CD45 showed a significant increase in the infiltration of immune cells in vaginal samples from LET group, with majority of cells localized to sub‐SSE (Figure 6A). There were 175 ± 38 vs 77 ± 17 CD45+ cells/mm^2^ (*P* < 0.05) in the sub‐SSE space of vaginal tissue samples from LET‐treated patients vs No‐LET controls. Muscularis, by contrast, had much fewer cells in LET‐treated vs control vaginal samples.

As protein expression analysis showed a significant increase in concentration of specific chemokines capable of attracting CD68+ macrophages, we quantified the infiltration of these cells into the tissues. We found a significant increase (*P* < 0.05) in the number of macrophages in vaginal biopsies of LET‐treated POP patients, particularly in the sub‐SSE layer (145 ± 54 vs 33 ± 5 CD68+ cells/mm^2^); and muscularis (70 ± 7 vs 31 ± 6 CD68+ cells/mm^2^) (Figure 6B).

## 4 DISCUSSION

Pelvic organ prolapse is a condition that affects millions of women worldwide and significantly disrupts their quality of life. With the POP incidence rates expected to increase with an ageing population, and the high reoccurrence rates of this condition,^2^ it is important to find therapeutic options that will help patients to enhance pelvic support. It has been established earlier that the strength of pelvic floor depends on the ECM components of connective tissue.^26^ Importantly, the symptoms of POP worsen with the decrease in circulating oestrogens, implying that connective tissue of pelvic floor weakens due to an age‐related oestrogen deprivation.^27^, ^28^ The weak pelvic floor is unable to provide proper support to pelvic organs, leading to the development of POP in older women.^6^ Thus, this study aims to examine whether LET influences connective tissue contents of the pelvic floor tissue in post‐menopausal women with severe grade POP.

While beneficial effects of post‐operative LET has been proven, there is no set guideline for the pre‐operative application of LET in POP patients due to insufficient clinical data.^29^ The benefits of pre‐operative vaginal oestrogen therapy show easier tissue handling, cure of several concomitant symptoms and improved long‐term results of surgery if oestrogen is continued post‐operatively.^4^ Recent randomized trials have shown that there is a benefit of using LET pre‐operatively in POP patients as it decreases degradative enzyme activity, increases collagen content and thickness of the vaginal wall thus improving the substrate for suture placement at time of surgical repair and maintaining connective tissue integrity of pelvic floor.^3^ However, there is still a need to examine the effect of LET on molecular levels to support its use on patients with POP as a guideline amongst urogynaecology professionals.

Previous research has demonstrated that the expression of ECM proteins (collagen I and III^30^ and elastin^31^) is altered in patients with POP as compared to asymptomatic patients with normal pelvic floor support. Additionally, the skin collagen content decreases by 2% in post‐menopausal women and this decrease correlated with oestrogen deficiency rather than with age,^33^ while the decrease in skin elastin content is age dependent^34^ with elasticity decreasing by 1.5% per year.^35^ Earlier studies have observed that following LET treatment there was an increase in skin collagen content,^37^ thickening of elastic fibres in the dermis, increase in their numbers and improvement of orientation.^38^ In accordance with previous studies, our current results show that LET can revert the menopause‐related ECM changes in vaginal skin by significantly improving collagen and elastin protein content in vagina of post‐menopausal POP patients, restoring vaginal tissue strength and elasticity. It has been also reported that in patients with POP, there are lower levels of elastin scaffold protein fibrillin‐1 and elastin binding protein, fibulin‐5, in the peri‐urethral tissue and uterosacral ligament.^25^, ^36^ We reported recently that patients with severe POP produce lower levels of maturation enzymes BMP‐1 and ADMATS2 as compared to healthy controls, indicating a decrease in ECM quality in POP patients and an impair of the functional integrity of the pelvic floor connective tissue.^39^ Current study demonstrates an increase in the *BMP1* gene expression following hormonal application in post‐menopausal women which may improve the quality of vaginal ECM.

The role of MMPs in pelvic floor disorders is well established. MMP2 and 9 (gelatinases), MMP1, 8 and 13 (collagenases) degrade collagen fibres; the stromelysins (MMP3 and 10) and related MMPs (MMP7 and 12) are capable of degrading elastin, cell adhesion molecules, proteoglycans, fibronectin and laminin, suggesting their involvement in modifying the vaginal ECM. Multiple studies have shown that POP is associated with increase in the expression of MMP1,^40^ MMP2 and MMP9^32^, ^41^ and decreases the activity of TIMP1‐4 leading to tissue degradation.^25^, ^40^ In our study, application of LET resulted in a decrease in the protein expression levels of MMP1, MMP2 and MMP3 an increase in TIMP1 and TIMP4. This is in agreement with other reports which showed that HRT inhibited the expression and secretion of MMP2 and MMP9 in human^42^ and improved the balance of TIMP‐MMP.^43^ Our studies clearly indicate that oestrogen limits undesirable ECM destruction by major degradation enzymes (MMP1, 2, 3), while not affecting the others (MMP7‐10, 12, 13) which supports the strengthening of pelvic floor tissue in post‐menopausal women.

Previous studies have shown that the immune system supports tissue homeostasis by regulating the expression of ECM components, ECM‐modulating enzymes and growth factors.^44^ Oestrogen is well known to exert its effect on the immune system through interaction with ESRs present on immune cells.^14^, ^46^ Abnormalities in hormonal status (such as deficiency in sex hormone) can make women susceptible to various immunologic impairments.^21^ Similarly, the oestrogen deprivation accompanying menopause is known to cause changes in the immune system called immunosenescence. One of the biggest age‐related adaptation that accompany immunosenescence is the involution of the thymus, resulting in fewer naïve and functionally deficient T cells, B cells and macrophages produced.^47^, ^48^, ^49^ Studies showed that low levels of oestrogen in post‐menopausal women can mitigate immune responses and make them susceptible to diseases and infections.^21^ There is a systemic increase in pro‐inflammatory serum markers, IL‐6, IL‐1b and TNF‐α in women after menopause that act as predictive markers of functional disability, fragility and mortality.^50^ This increase causes a continuous stimulation of the immune system resulting in a subclinical inflammatory status known as inflammaging.^51^

The effect of increasing age on type 1 (Th2) and type 2 (Th2) cytokines is challenging to study as it can be altered by many factors. Majority of studies detected increased IFN‐γ secretion by activated CD4+ and CD8+ lymphocytes^52^, with the ratio of IFN‐γ/IL‐4 and IFN‐γ/IL‐10 both significantly decreased in the older patients indicating an age‐related shift from Th1 to Th2 cytokine profiles.^53^ These lymphocytes can participate in immunoregulation, tissue remodelling, eosinophil accumulation as well as induce strong antibody responses, while inhibiting several functions of phagocytic cells resulting in phagocyte‐independent inflammation. On the other hand, Th1 subsets evoke cell‐mediated immunity and phagocyte‐dependent inflammation and also have tumour suppressive activity.^54^ Here, we observed an increase in IFN‐γ following LET which suggests that the lymphocytes attracted to vaginal tissue might express Th1 cytokines. For instance, Yu et al showed that increased Th1 activity directly enhances both LOX and cross‐linked collagen.^44^ Shi et al^45^ also demonstrated that lymphocytes may induce tissue fibrosis by stimulating the release of TGF‐β and regulating LOX expression in the heart. Therefore, it is possible that the immune cells also support tissue homeostasis.

Thus, we have tested first the basal expression of 44 soluble cytokines, within vaginal tissue samples of post‐menopausal women with severe POP. The exact source of these vaginal cytokine is unknown. Kumru et al measured the levels of various cytokines in women following surgical menopause and found evidence of immunodeficiency due to a decrease in the CD19+ B cell subpopulation, with simultaneous increase in CD8+ cells and reduced CD4/CD8 cell ratio.^55^ Studies have shown that in addition to leucocytes, fibroblasts are capable of eliciting an immune response by direct communication with immune cells and by modulating the release of cytokines.^56^ The effect of pelvic floor disorders on the cytokine production is not fully understood. A previous study found an increase in the expression levels of IL‐6, CCL2, CXCL1, CXCL2 and CXCR4 gene in patients with POP compared to asymptomatic controls.^57^ Zhao et al^58^ observed an increase in the expression of IFN‐γ and its receptors IFNGR1 and IFNGR2 in vaginal tissue of pre‐menopausal women with POP, suggesting that there is an association of inflammatory cytokines (i.e. IFN‐γ) and POP. It is however not clear whether cytokines play a regulatory role in POP development or they are a consequence of chronic inflammatory response caused by tissue prolapse.

Next, we examined the effect of LET on vaginal cytokines in post‐menopausal women with severe POP and recorded an increase in 14 cytokines all capable of mediating pro‐inflammatory responses. We suggested that these chemokines may induce chemotaxis of monocytes/macrophages (MIP1b, IFN‐γ, IL16, IP‐10, CCL2, CCL3, CCL8, CXCL12, RANTES), T cells (IL16, IP‐10, CCL2, CCL8, CXCL12), B cells (CXCL13, CXCL12, CCL19) and granulocytes (IL16, CCL8) in human vaginal tissue following LET, while G‐CSF is involved in proliferation and differentiation of haematopoietic precursors of granulocytes and in production of other cytokines in vivo (i.e. TNF‐α).^59^ Using a pan‐leucocyte marker CD45, we confirmed that there was a significant increase in the total number of leucocytes infiltrating to vagina, in particular, an increase in the infiltration of CD68+ macrophages in the sub‐epithelial and muscularis layers. A few studies have reported the presence of inflammatory cells in the levator ani muscle and vaginal tissue of women with POP.^25^, ^40^ Earlier we observed co‐expression of LOXL1 in CD68+ macrophages infiltrating vaginal tissue of pre‐menopausal women with POP.^41^

Based on the vaginal cytokine profile, we speculate that the invading immune cells are a mix of both major macrophage phenotypes, M1 (which elucidates type 1 inflammation response and antimicrobial activity), and M2 (which plays a role in ECM deposition). We propose that this influx might increase protection against pathogenic vaginal microbiota, as well as strengthening of the ECM.^53^ Similar to our results, previous studies of immune system response to HRT observed an increase in TNF‐α,^23^ M‐CSF,^24^ IL‐6,^60^ and IFN‐γ^55^ as well as induction of both M1 and M2 phenotypes of macrophage.

## 5 CONCLUSION

There has been discussion about the efficacy of LET as a potential supplemental agent for post‐menopausal POP patients improving the quality of their pelvic floor. The data presented here demonstrate an increase in the amount of structural proteins, collagen and elastin, up‐regulation of their synthesis enzyme, coupled with a decrease in their degradation enzymes, suggesting a strengthening effect of oestrogen on the pelvic tissue ECM. In addition, our results show that LET plays an important role in the activation of immune system within the local vaginal environment, therefore resisting menopause‐related changes and in improving urogenital tract tissue regeneration. Moreover, several studies demonstrate that low‐dose LET improves vaginal symptoms in women with history of breast cancer with plasma estradiol levels remained in the normal range for post‐menopausal women.^61^ Altogether, our results show that LET plays a protective role in pelvic tissue by limiting the undesirable effects of age and hypoestrogenism and relieving the GSM symptoms in post‐menopausal women affected by POP.

## CONFLICTS OF INTEREST

The author(s) declared no potential conflicts of interest with respect to the research, authorship, and/or publication of this article.
