# Supplementary figures and images for: Local oestrogen therapy modulates extracellular matrix and immune response in the vaginal tissue of post‐menopausal women with severe pelvic organ prolapse
Source: J Cell Mol Med. 2019 Feb 17;23(4):2907–19. doi: 10.1111/jcmm.14199 (PMC6433658; doi:10.1111/jcmm.14199)

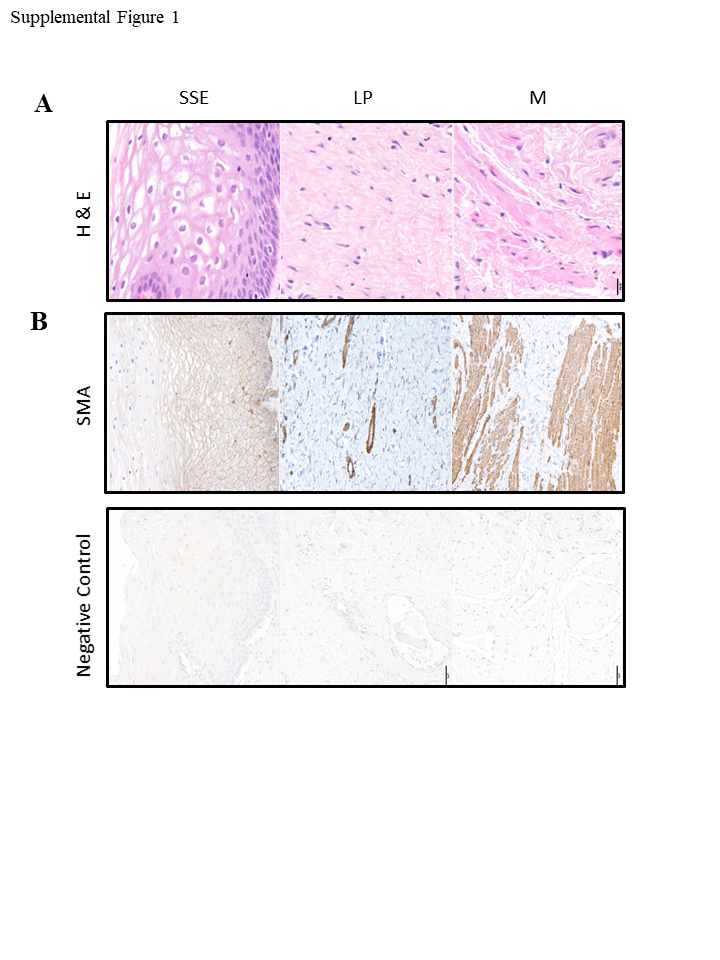

Supplement: Supplementary file 1 [file JCMM-23-2907-s001.tif]

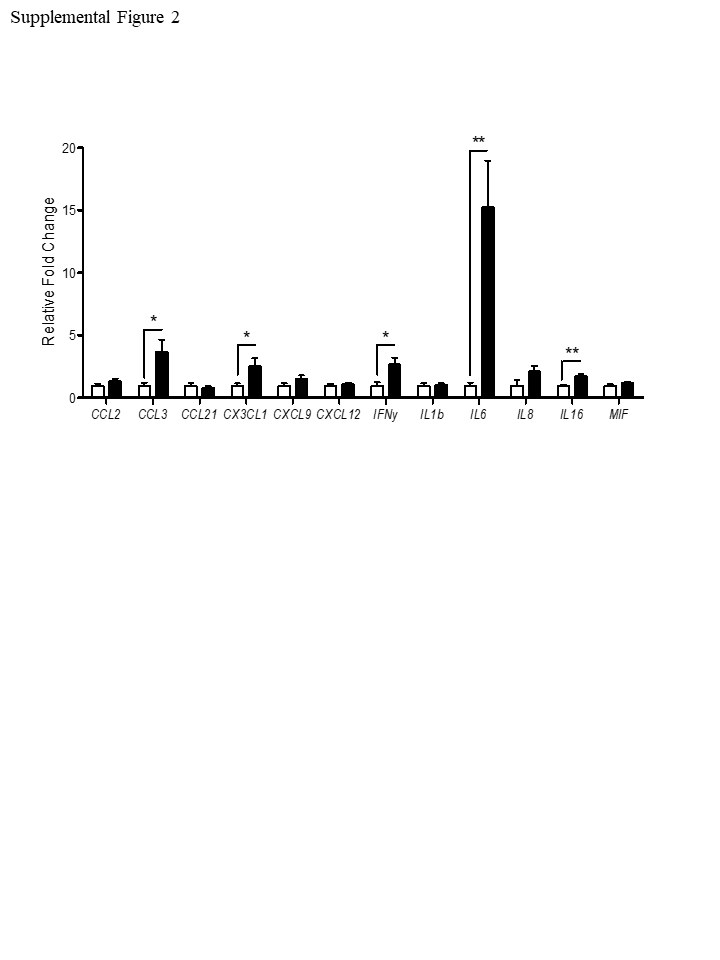

Supplement: Supplementary file 2 [file JCMM-23-2907-s002.tif]
