# Supplementary material for: Local oestrogen therapy modulates extracellular matrix and immune response in the vaginal tissue of post‐menopausal women with severe pelvic organ prolapse
Source: J Cell Mol Med. 2019 Feb 17;23(4):2907–19. doi: 10.1111/jcmm.14199 (PMC6433658; doi:10.1111/jcmm.14199)
Supplement: Supplementary file 3 [file JCMM-23-2907-s003.docx]

**Supplemental Table 1:7 The standard range and sensitivities of tested cytokines using Luminex assay**

40-plex

| **Symbol** | **Name** | **Assay Standard Range**  **(pg/ml)** | **Sensitivity**  **(pg/ml)** |
| --- | --- | --- | --- |
| CCL1 | Chemokine (C-C motif) Ligand 1 | 2.2- 35345 | 1.6 |
| CCL2 | Chemokine (C-C motif) Ligand 2 | 0.2- 3668 | 0.1 |
| CCL3 | Chemokine (C-C motif) Ligand 3 | 0.3- 4433 | 0.3 |
| CCL7 | Chemokine (C-C motif) Ligand 7 | 1.5- 23898 | 1.3 |
| CCL8 | Chemokine (C-C motif) Ligand 8 | 0.3- 4355 | 0.04 |
| CCL11 | Chemokine (C-C motif) Ligand 11 | 1.7- 27354 | 2.5 |
| CCL13 | Chemokine (C-C motif) Ligand 13 | 0.2- 2896 | 0.1 |
| CCL15 | Chemokine (C-C motif) Ligand 15 | 1.5- 24284 | 0.2 |
| CCL17 | Chemokine (C-C motif) Ligand 17 | 1.4- 23374 | 1.1 |
| CCL19 | Chemokine (C-C motif) Ligand 19 | 2.3- 37312 | 1.1 |
| CCL20 | Chemokine (C-C motif) Ligand 20 | 0.6- 9286 | 0.1 |
| CCL21 | Chemokine (C-C motif) Ligand 21 | 2.6-42091 | 12.0 |
| CCL22 | Chemokine (C-C motif) Ligand 22 | 1.1- 18233 | 0.5 |
| CCL23 | Chemokine (C-C motif) Ligand 23 | 1- 15964 | 0.23 |
| CCL24 | Chemokine (C-C motif) Ligand 24 | 1.04- 16995 | 3.2 |
| CCL25 | Chemokine (C-C motif) Ligand 25 | 6.3- 102616 | 4.9 |
| CCL26 | Chemokine (C-C motif) Ligand 26 | 0.9- 14324 | 0.5 |
| CCL27 | Chemokine (C-C motif) Ligand 27 | 1.2-19445 | 3.4 |
| CXCL1 | Chemokine (C-X-C motif) Ligand 1 | 2.9- 46851 | 6.3 |
| CXCL2 | Chemokine (C-X-C motif) Ligand 2 | 1.2- 19090 | 2.7 |
| CXCL5 | Chemo5ine (C-X-C motif) Ligand 6 | 13.2- 216942 | 5.7 |
| CXCL6 | Chemokine (C-X-C motif) Ligand 6 | 1 - 15719 | 0.6 |
| CXCL9 | Chemokine (C-X-C motif) Ligand 9 | 5.6- 92565 | 1.2 |
| CXCL11 | Chemokine (C-X-C motif) Ligand 11 | 1.5- 25273 | 0.05 |
| CXCL12 | Chemokine (C-X-C motif) Ligand 12 | 9.9- 161788 | 10.3 |
| CXCL13 | Chemokine (C-C motif) Ligand 13 | 0.3-5641 | 0.1 |
| CXCL16 | Chemokine (C-X-C motif) Ligand 16 | 0.3- 5329 | 0.1 |
| CX3CL1 | chemokine (C-X3-C motif) ligand 1 | 4.35- 71290 | 0.9 |
| GM-CSF | Granulocyte-Macrophage Colony Stimulating Factor | 2.25- 36901 | 0.2 |
| IFN-γ | Interferon gamma | 0.15- 2393 | 6.4 |
| IL1b | Interleukin 1 beta | 3.6- 58216 | 0.6 |
| IL2 | Interleukin-2 | 0.4- 6182 | 1.6 |
| IL4 | Interleukin-4 | 0.6- 10332 | 0.7 |
| IL6 | Interleukin-6 | 1.1- 18143 | 2.6 |
| IL8 | Interleukin-8 | 0.7- 11342 | 1.0 |
| IL10 | Interleukin-10 | 1.8- 28901 | 0.3 |
| IL16 | Interleukin-16 | 1.6- 26246 | 0.4 |
| IP-10 | Interferon gamma-induced protein 10 | 0.3- 5522 | 1.1 |
| MIF | Macrophage migration Inhibitory Factor | 20.5- 336202 | 1.5 |
| TNF-α | Tumor necrosis factor alpha | 0.8- 13398 | 6.0 |

5-plex

| **Symbol** | **Name** | **Assay Standard Range**  **(pg/ml)** | **Sensitivity**  **(pg/ml)** |
| --- | --- | --- | --- |
| G-CSF | Granulocyte colony-stimulating factor | 1.7-28267 | 1.7 |
| IL-1Ra | Interleukin-1 Receptor antagonist | 7.4-121425 | 5.5 |
| MIP-1b | Macrophage inflammatory protein-1beta | 0.3-4543 | 2.4 |
| RANTES | Regulated and normal T cell expressed and secreted | 0.9-14838 | 1.8 |
| VEGF | Vascular Endothelial Growth Factor | 2.4-38806 | 3.1 |
