# Supplementary material for: Local oestrogen therapy modulates extracellular matrix and immune response in the vaginal tissue of post‐menopausal women with severe pelvic organ prolapse
Source: J Cell Mol Med. 2019 Feb 17;23(4):2907–19. doi: 10.1111/jcmm.14199 (PMC6433658; doi:10.1111/jcmm.14199)
Supplement: Supplementary file 4 [file JCMM-23-2907-s004.docx]

| **Cytokines** | **Concentration (pg/mg)** |
| --- | --- |
| MIF | 82770.4 ± 10013.3 |
| CCL21 | 4066.8 ± 537.7 |
| IL16 | 1972 ± 193.4 |
| RANTES | 1217.7 ± 192.9 |
| VEGF | 1170.8 ± 200.3 |
| CXCL9 | 372.1 ± 90.4 |
| CX3CL1 | 264.1 ± 17 |
| CCL25 | 169.3 ± 6.1 |
| MIP-1b | 105.4 ± 19 |
| CCL23 | 85.4 ± 13.9 |
| CCL15 | 75.4 ± 22.2 |
| CCL22 | 68.1 ± 13.4 |
| CCL24 | 65.9 ± 16.2 |
| CXCL16 | 49.8 ± 7.3 |
| CCL2 | 40.1 ± 5.2 |
| CCL27 | 32.8± 3.6 |
| CXCL1 | 30 ± 1.9 |
| CCL26 | 26.3 ± 5.6 |
| IL8 | 22.9 ± 8.5 |
| CXCL12 | 19.1 ± 3.5 |
| GM-CSF | 17 ± 0.4 |
| CXCL2 | 15 ± 2.8 |
| CCL11 | 11.1 ± 0.5 |
| G-CSF | 10.7 ± 2.6 |
| IL1b | 6.9 ± 1.0 |
| CCL1 | 5.9 ± 0.5 |
| CCL19 | 5.2 ± 0.7 |
| CCL17 | 4.2 ± 2.2 |
| CCL7 | 4.2 ± 0.3 |
| CCL13 | 4.2 ± 0.7 |
| CXCL11 | 4.1 ± 1.9 |
| IL10 | 2.8 ± 0.5 |
| CXCL6 | 2.45 ± 0.8 |
| CCL3 | 2.45 ± 0.7 |
| CCL8 | 2.1 ± 0.5 |
| IL6 | 2.1 ± 0.7 |
| TNFa | 1.6 ± 0.2 |
| IP-10 | 1.1 ± 0.2 |
| IL4 | 0.9 ± 0.4 |
| CCL20 | 0.8 ± 0.2 |
| IL2 | 0.6 ± 0.3 |
| CXCL13 | 0.4 ± 0.1 |
| IFNY | 0.1 ± 0.02 |
